# Application of 3D Printing Technology in Increasing the Diagnostic Performance of Enzyme-Linked Immunosorbent Assay (ELISA) for Infectious Diseases

**DOI:** 10.3390/s150716503

**Published:** 2015-07-08

**Authors:** Harpal Singh, Masayuki Shimojima, Tomomi Shiratori, Le Van An, Masami Sugamata, Ming Yang

**Affiliations:** 1 Department of Intelligent Mechanical Systems, Graduate School of System Design, Tokyo Metropolitan University, 6-6 Asahigaoka, Hino, Tokyo 191-0065, Japan; E-Mails: singhha3@gmail.com (H.S.); tom7032@gmail.com (T.S.); 2 Department of Virology 1, National Institute of Infectious Diseases, 4-7-1 Gakuen, Musashimurayama, Tokyo 208-0011, Japan; E-Mail: shimoji-@nih.go.jp; 3 Department of Microbiology, Hue University of Medicine and Pharmacy, 6 Ngo Quyen St., Hue 47000, Vietnam; E-Mails: levanan.hump@gmail.com (L.V.A.); bmsasugatmu@gmail.com (M.S.); 4 Department of Hygiene and Public Health, Graduate School of Human Health Sciences, Tokyo Metropolitan University, 1-1 Minami-Osawa, Hachioji, Tokyo 192-0397, Japan

**Keywords:** infectious diseases, 3D printing, rapid diagnostics, ELISA

## Abstract

Enzyme-linked Immunosorbent Assay (ELISA)-based diagnosis is the mainstay for measuring antibody response in infectious diseases and to support pathogen identification of potential use in infectious disease outbreaks and clinical care of individual patients. The development of laboratory diagnostics using readily available 3D printing technologies provides a timely opportunity for further expansion of this technology into immunodetection systems. Utilizing available 3D printing platforms, a ‘3D well’ was designed and developed to have an increased surface area compared to those of 96-well plates. The ease and rapidity of the development of the 3D well prototype provided an opportunity for its rapid validation through the diagnostic performance of ELISA in infectious disease without modifying current laboratory practices for ELISA. The improved sensitivity of the 3D well of up to 2.25-fold higher compared to the 96-well ELISA provides a potential for the expansion of this technology towards miniaturization and Lab-On-a-Chip platforms to reduce time, volume of reagents and samples needed for such assays in the laboratory diagnosis of infectious and other diseases including applications in other disciplines.

## 1. Introduction

Techniques for enzyme labeling of antibodies, more commonly known as Enzyme-Linked Immunosorbent Assay (ELISA) is the method of choice used in various diagnostics and research laboratories for the demonstration of immunologic reactions [1,2,3,4]. These resulting labeled antibody conjugates have become the mainstay in the measurement of antibody levels in infectious diseases and among the methods used in the identification of infectious disease pathogens [1,5,6].

At present, most ELISAs are performed in 96-well microtiter plates which are made of polystyrene (PS). Techniques to reliably immobilize antigens or antibodies on PS are responsible for the immunosorbent/solid phase reactions and the overall sensitivity of the 96-well ELISA [2,3,4]. Although much progress has been made (96-well plate ELISA) in terms of its automation (e.g., simplicity in performance) and ability to perform multiple assays at a time, numerous limitations still exist such as the time (e.g., several hours) required to complete the assay, large sample and reagent volume requirements and the fact they must be carried out in a laboratory [2,4,5,6,7,8,9].

Faced by these challenges, many newer platforms based on micro total analysis systems (µ-TAS) or simply miniaturization have been developed aimed at increasing the surface area available for reaction, improving reaction kinetics and delivery and flow of samples and reagents. Various innovations have provided enhancements in detection systems, increased automation and handling of samples, ensuring high-throughput, time- and cost-efficiency and improved sensitivity compared to the traditional ELISA. Some of these innovations include inkjet-driven micro-droplet reaction systems [7,8,9,10,11], Lab-On-a-Chip (LOC) [4,12], flow-based analytical systems [13], sequential injection analysis (SIA) [14]- and surface plasmon resonance (SPR)-based devices [15] incorporating the use of microbeads [7,8,9,10,11,12,13,14], carbon nanotubes [4,16], a variety of non-traditional solid-phase material composition and fabrication technologies [7,9,13,17,18,19]. Although many of these newer technologies have been validated, they need sophisticated equipment, require complex fabrication technologies, are expensive and complex to use and are unmatched in terms of the number of samples that can be tested simultaneously as the traditional ELISA [4]. The uptake of these platforms may be low since long established institutional practices centered on the use of traditional ELISA will need to be completely overhauled, which is a factor this study aimed to avoid.

The introduction of 3D printing technology in the 1980s has found application in numerous disciplines, including laboratory medicine and has had a substantial impact in the fabrication of miniaturized biochemical detection systems such as microfluidics and LOC technologies [20]. The development of the Standard Tessellation Language or STereoLithography (.STL) file format as a transmission link between the computer aided design (CAD) software and 3D printers allows the rapid creation of prototypes and quick readjustment to be made following prototype assessments [20,21,22]. Various 3D printing platforms, based on Fused Deposition Modeling^®^ (FDM) technologies exist that make use of polymer-based materials such as acrylonitrile-butadiene-styrene (ABS), polycarbonate (PC), polyphenylsulfone (PPSF/PPSU) and other thermoplastics that are cheap, easy to fabricate and are disposable, all important aspects in various immunodetection systems [21,22,23,24,25,26]. The potential of such newer fabrication technologies in the development of diagnostics and application in laboratory medicine must not be excluded [27,28]. 

In this study, we report the first trial of 3D printing technology in the diagnostic performance of ELISA as well as the structural characteristics and validation in the immunological diagnosis of infectious diseases and the future expansive potential of this technology.

## 2. Experimental Section 

### 2.1. Design Rationale

**Figure 1:**
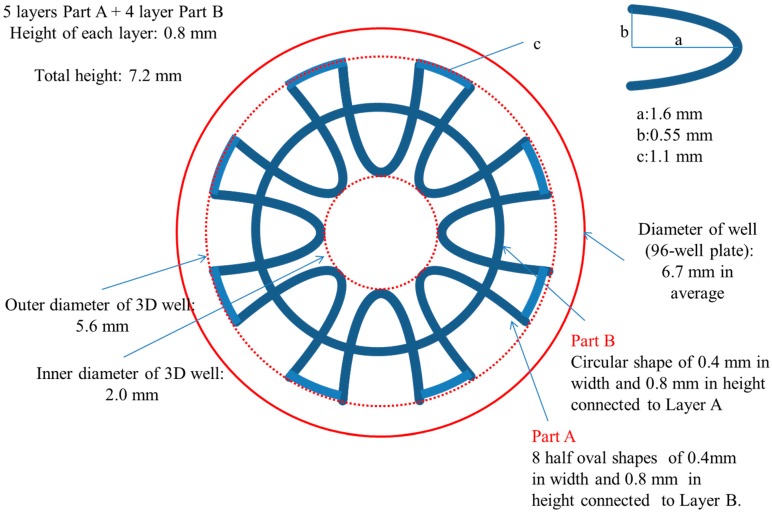
Schematic diagram of the 3D well (top view).

The development of the prototype design used in this study, which we named ‘3D well’, aimed at increasing the surface area available for reaction and decreasing the diffusion distance to shorten the reaction time compared to the 96-well plate used in traditional ELISAs. Additionally, the 3D well which is patent on both ends was designed to fit snuggly into the wells of the 96-well plate. The utilization of the 3D wells placed inside 96-wells was aimed at providing support/base and not to contribute to the ELISA efficiency of the 3D well. This was an important consideration to allow multiple assays to be carried out simultaneously and to maintain the standard practices of 96-well ELISA methods. Briefly, the 3D well was designed composed of two parts (A and B) of two different shapes. Part A (outer, five layers in total, symmetrically distributed 8-half oval shapes) and B (inner, four layers in total, circular in shape) piled up alternatively. This was important to enable the 3D well to be printed in one-step. The total surface area was 651 mm^2^ [*vs*. surface area of well (96-well plate): 151 mm^2^]. A schematic diagram showing the parameters used in the designing of the 3D well is shown in Figure 1. The 3D well was drafted using the CAD software and converted to an .STL file using the inbuilt Insight 10.2™ job processing and management software (Stratasys^®^, Eden Prairie, MN, USA). 

### 2.2. Fabrication Technology

The 3D well was printed using a Fortus 250mc (Stratasys^®^) 3D printer using ABS*plus*-P430™ (acrylonitrile-butadiene-styrene) as build material and soluble release material (SR-30 Soluble Support) composed of a terpolymer of methacrylic acid, styrene and butylacrylate. Briefly, the Fortus 250mc incorporates a 10 × 10 × 12 inch (254 × 254 × 305 mm, XYZ) build envelope and three print material layer thicknesses of 0.007, 0.010 and 0.013 inch (0.178, 0.254 and 0.330 mm). This study utilized the 0.178 mm layer thickness. The system specifications of the Fortus 250mc 3D printer, process parameters and materials used in this study are summarized in Table 1. 

The printing of the prototype was performed through an extruded (X-Y platform) deposition of semi-molten layers (300 °C) composed of ABS*plus*-P430™ and SR-30 Soluble Support, alternatively from bottom up (Z stage), beginning first with the support material to create a base. The support material was then removed through sonication in a water-based detergent solution at 30 °C for 1–2 h. 

**Table 1:** System configuration of Fortus 250mc (Stratasys^®^, Eden Prairie, MN, USA) printer, process parameters and materials used in this study.

**System Configuration**	
Build Envelope (XYZ):	254 × 254 × 305 mm (10 × 10 × 12 inch)
Layer Thicknesses:	0.007 inch (0.178 mm)
System Size/Weight:	838 × 737 × 1143 mm (33 × 29 × 45 inch)
Achievable Accuracy:	±0.241 mm (±0.0095 inch)
**Material**	
Build Material:	ABS*plus*-P430™ (Acrylonitrile-butadiene-styrene)
Soluble Release Material:	*SR-30 Soluble Support* (terpolymer of methacrylic acid, styrene and butylacrylate)
**Process Parameters**	
Internal Temperature:	85 °C
Modeling Head Temperature:	300 °C
Maximum Scanning Speed:	91.44 mm/s

### 2.3. Surface Modification

The 3D well was made hydrophilic by a chemical etching treatment procedure employed in this study [29]. In preparation for chemical etching, physical adsorbents present in the 3D wells were removed and degreased by sonication for 3 min at 50 °C in a water bath followed by removal of the release agents using a surfactant solution. Then, chemical etching was carried out on each 3D well in an etching solution composed of (CrO_3_ 380 g + H_2_SO_4_ 370 g)/L at 65 °C for 3 min. 

### 2.4. Sample Collection and Ethical Statement 

In an ongoing collaboration for the serological monitoring of infectious diseases in Central Vietnam, a total of 272 serum samples from patients who visited the Outpatient Department of the Hue University Hospital between March and June 2014 were collected and screened for *Rubella virus* antibodies by immunoglobulin G (IgG) ELISA (96-well ELISA). Two samples, serum containing *Rubella virus* antibody (*Rubella virus* antibody positive sample) and serum not containing *Rubella virus* antibody (*Rubella virus* antibody negative sample), one each respectively, were selected and subsequently used in the 3D well ELISA. Serum samples collected from all patients were carried out under informed consent. All protocols and procedures were approved by the Research and Ethical Committee for the use of human subjects of the Hue University of Medicine and Pharmacy. 

### 2.5. IgG ELISA

IgG ELISA was carried out using treated and untreated 3D wells and subsequently for validation (by titration) purposes. For confirmation purposes, parallel testing by 96-well ELISA was carried out at every step. Briefly, 3D wells and 96-well ELISA plates (Sigma-Aldrich, St. Louis, MO, USA) were coated with 100 µL of predetermined optimal quantity (1:2) of *Rubella virus* Hemaglutination Antigen (HA) (Denka-Seiken, Tokyo, Japan) in sodium carbonate buffer at 4 °C overnight. Blocking was performed with 200 µL of 0.05% Tween-20 phosphate buffer solution (PBS-T) containing 5% skimmed milk (PBST-M), followed by incubation for 1 h at room temperature (RT).

Primary antibodies consisting of 100 µL of test samples and reference antiserum (positive and negative, Denka-Seiken) used at a 1-point dilution of 1:400 was added to each well followed by incubation for 1 h at RT. The positive and negative reference antiserum was used to confirm the specificity of the binding and to determine the positive and negative cut-off values. For validation purposes only treated 3D wells and 96-well plates were used with primary antibodies consisting of 100 µL of test samples were diluted in PBST-M four-folds from 1:100 to 1:6400. This titrated mixture was added to each well and incubated for 1 h at RT. 

Horseradish peroxidase (HRP, 1:1000, 100 µL) of conjugated goat anti-human IgG (Invitrogen, Camarillo, CA, USA) diluted in PBST-M which served as the secondary antibody was added to each well and followed by incubation for 1 h at RT. Finally, substrate solution (100 µL) containing 2,2′-azino-bis(3-ethylbenzthiazoline sulfonic acid) (ABTS) solution (Roche Diagnostics, Mannheim, Germany) was added to each sample well. The wells were incubated for 30 min at room temperature and optical density at 405 nm (OD_405_) was measured against a reference of 490 nm using a Model 680 Microplate Reader (Bio-Rad, Hercules, CA, USA). 

Three rounds of washing were performed in between all steps with 300 µL of PBS-T per round. In the 3D well ELISA, each step was carried out by placing the 3D well in a new well of a 96-well plate as support/base and to neglect the ELISA efficiency contribution from the 96-well plate. The 3D well structure was removed carefully following incubation with the substrate solution before OD_405_ measurement. A summary of the IgG ELISA protocol used in this study in presented in Table 2. 

**Table 2:** IgG ELISA protocol for the detection of *Rubella virus* antibodies in human serum samples used in this study (3D well and 96-well, ELISA).

**Step 1: Coating** 100 µL of *Rubella virus* HA antigen (1:2) in sodium carbonate buffer Incubation at 4 °C overnight
**Washing:** three rounds of washing with 300 µL of PBS-T per round
**Step 2: Primary Antibody** 100 µL of test samples, positive and negative reference antiserum diluted (1:100, 1:400, 1:1600 and 1:6400) in PBST-M; Incubation at room temperature (RT), 1 h.
**Washing:** three rounds of washing with 300 µL of PBS-T per round
**Step 3: Secondary Antibody** 100 µL of goat anti-human IgG – horseradish peroxidase (HRP) conjugate diluted in PBST-M (1:11,000); Incubation at RT, 1 h
**Washing:** three rounds of washing with 300 µL of PBS-T per round
**Step 4: Substrate** 100 µL of 2,2′-azino-bis-(3-ethylbenzthiazoline sulfonic acid) (ABTS) solution Incubation at RT, 30 min **Absorbance Measurement** Optical density at 405 nm (OD_405_) was measured against a reference of 490 nm

## 3. Results

### 3.1. Physical Evaluation 

The 3D well consisted of a total of nine layers made up of: (1) five 8-half oval shaped layers (which include the top- and bottom-most layer) that are distributed around a central core. These layers are connected above and/or below with (two) four circular shaped layers. 

**Figure 2:**
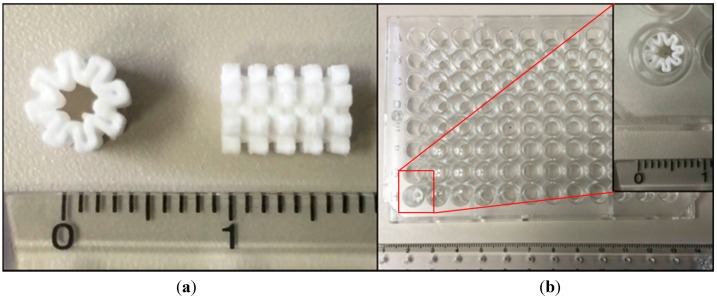
3D well prototype showing (**a**) top-most layer composed of 8-half oval shapes (left) and side view showing 5 layers of Part A interspersed by 4 layers of Part B (circular shape) (right) and (**b**) placement of 3D well in the 96-well plate.

These layers form the 3D well in a closed-wall, patent at both ends and share a common core structure. Figure 2a shows the actual 3D well and the placement of the 3D well in the 96-well plate is shown in Figure 2b. In this 3D well, the inner diameter (core) is 2.0 mm, the outermost diameter is 5.5 mm [well diameter, 96-well plate: top (7.0 mm); bottom (6.2 mm)] and the total height is 7.2 mm (inside depth of well, 96-well plate: 11.4 mm) comparable to the parameters established in the 3D well designing stage. Measurements are averages taken from three 3D wells and the error in dimensions is ±0.1 mm (±0.0039 inch). These results are consistent with the achievable accuracy of 3D printing platforms such as the one used in this study of ±0.241 mm (±0.0095 inch) [23]. 

### 3.2. Surface Modification in ELISA Efficiency

**Figure 3:**
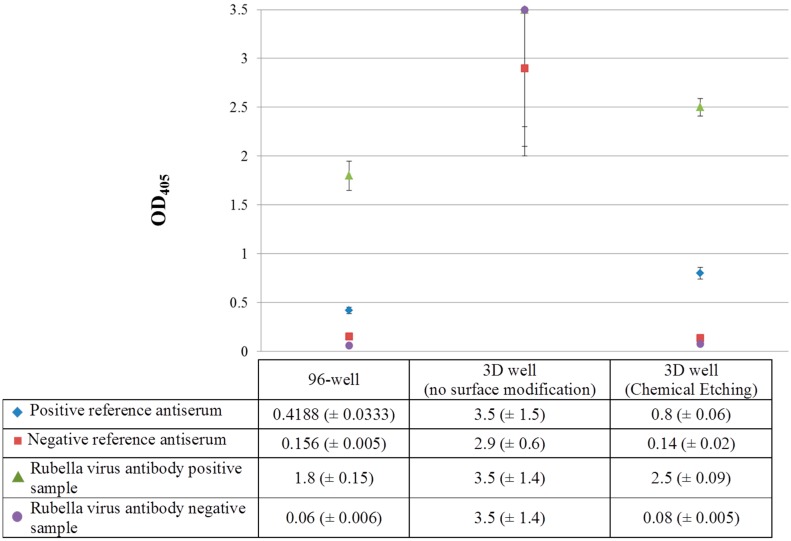
Surface modification and ELISA efficiency for *Rubella virus* antibody at 1-point dilution of primary antibody (1:400) (OD_405_: optical density measured at 405 nm).

Following the chemical etching method used in this study for modification of the 3D well to a hydrophilic surface, a 1-point dilution of primary antibody of 1:400 was carried out for IgG ELISA for *Rubella virus* antibody. These results, obtained from 3D wells following chemical etching, untreated 3D wells and 96-well plates are shown in Figure 3. 

Following surface modification by chemical etching, the *Rubella virus* antibody positive sample showed an OD value above the cutoff value of the positive reference antiserum (OD_405_: 2.5 *vs*. 0.8) while the *Rubella virus* antibody negative sample showed an OD value below the cutoff value of the negative reference antiserum (OD_405_: 0.08 *vs*. 0.14), respectively. Additionally, the 3D well treated by chemical etching showed a higher detection capacity for the *Rubella virus* antibody positive sample and positive reference antiserum compared to the 96-well ELISA [OD_405_: 2.5 and 0.8 (3D well) *vs*. 1.8 and 0.4 (96-well)] suggesting a 1.4- and 2-fold increase in sensitivity of the 3D well ELISA compared to the 96-well ELISA. Although the OD values for the *Rubella virus* antibody negative sample and negative reference antiserum for the 3D well (treated) and 96-well ELISA were comparable (OD_405_: 0.14 and 0.08 *vs*. 0.156 and 0.06), this suggests the absence of binding (specific or non-specific) in the *Rubella virus* antibody negative sample, thereby validating the specificity of the 3D well ELISA. In addition, the water contact angle for untreated and 3D wells treated by chemical etching was 111.8° and 41.3° respectively suggesting a modification of ABS by chemical etching to become hydrophilic (data not shown).

### 3.3. Validation

**Figure 4:**
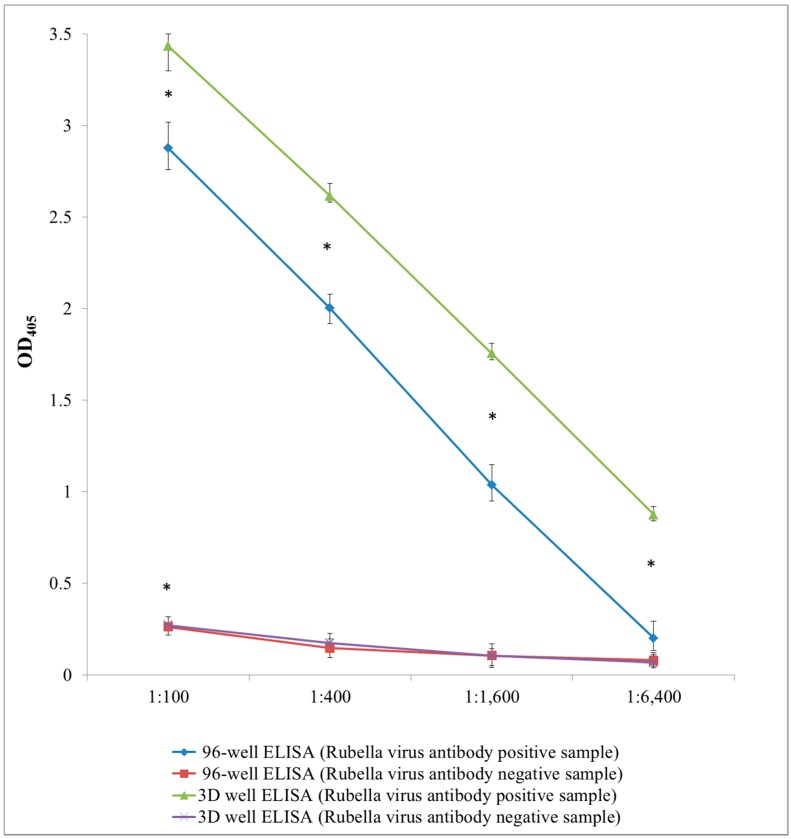
Validation by IgG ELISA for rubella virus antibody at 1:100 to 1:6400 dilution of primary antibody (OD_405_: optical density measured at 405 nm). Points represent mean of OD_405_ values of 6 tests (n = 6) and error bars represent range of OD_405_ data obtained. * Asterisk denominates statistical significance (Student’s t-test, *p* < 0.05).

The sensitivity of detection of *Rubella virus* antibodies in the *Rubella virus* antibody positive sample was higher using the 3D well following surface modification by chemical etching at all primary antibody dilutions used in the validation (1:100, 1:400, 1:1600 and 1:6400) compared to the 96-well [OD_405_ (3D well *vs*. 96 well): 1:100 (3.4 *vs*. 2.9); 1:400 (2.8 *vs*. 1.7); 1:1600 (1.6 *vs*. 0.9 and 1:6400 (0.9 *vs*. 0.4)]. The sensitivity of detection for the *Rubella virus* antibody positive sample using the 3D well ELISA was 1.2-fold (1:100), 1.6-fold (1:400), 1.7-fold (1:1600) and 2.25-fold (1:6400) (average: 1.7-fold) higher compared to the 96-well ELISA at each dilution point, respectively. Student’s t-test analysis showed that the sensitivity based on the OD_405_ values obtained for the *Rubella virus* antibody positive sample at all dilution points using the 3D well were higher than that of the 96-well (*p* < 0.05) (Figure 4). 

The OD values for the *Rubella virus* antibody negative sample and negative reference antiserum for the 3D well and 96-well ELISA were comparable at all serum dilution points used suggesting the absence of binding (specific or non-specific) in the *Rubella virus* antibody negative sample. These results were conclusive of the presence and absence of *Rubella virus* antibodies in the test samples, respectively (Figure 4). Since 3D wells were removed after incubation from the ABTS substrate solution placed in wells of the 96-well plate, loss of the ABTS substrate solution may not have been totally excluded. However, the signal variations observed at each dilution point using the *Rubella virus* antibody positive sample by the 3D well ELISA was smaller than that of the 96-well suggesting minimal loss of this solution (Figure 4).

The consistency and accuracy achieved in the production of the different 3D wells through 3D printing platforms and the suggested hydrophilicity of the ABS surface following chemical etching are critical components in the efficiency of the 3D well ELISA. OD_405_ was measured after 5 min incubation with the substrate solution containing ABTS in 3D wells due to an observable color change. This observation further supports the increased sensitivity of the 3D well compared to the 96-well plate. 

## 4. Discussion

3D printing technologies are widely used technologies for rapid prototyping due to its ease in design development to final product development [20,26]. The use of design development software such as CAD and numerous software to create accurate motion paths for 3D printing using readily available semi-molten polymers materials or its combinations under controllable environments and equipment to ensure design accuracy [20,21,22,23,25,26], provides room for quick assessments to be carried out and readjustments to be made. 

The rapid development of the 3D well design used in this study by increasing the surface area for reaction involving the inner and outer wall surfaces of the 3D well as a potential contributor to a more sensitive ELISA system allowed the rapid performance of an initial assessment (IgG ELISA) without the need of numerous optimization steps (e.g., reducing time and reagent volumes) and modifications to standard institutional ELISA practices to be made. Although this study did not use reduced volumes or time in the validation, the improved detection sensitivity achieved in this study with a reduction in the 3D well size compared to 96-well plates will enable future optimizations to be explored. Although the 3D wells developed in this study showed an improved sensitivity compared to the 96-well ELISA, the increased sensitivity may have been contributed by other factors such as an to an increased binding capacity of proteins to the treated material of the 3D well material (*i.e.*, ABS), which is a factor this study did not exclude. 

Although ABS is the most commonly used polymer material in one-step manufacturing such as FDM technology at present, the potential to utilize almost any thermoplastic polymer and the application of materials with different chemical properties in the 3D printing process must not be excluded [21], thus providing opportunities for the use of hydrophilic materials that can be readily used to develop vessels for ELISAs in the future. Although beyond the scope of this study, scanning electron microscopy (SEM) images obtained following chemical etching showed no significant difference in the ABS surface of different 3D wells carried out at the same time. This is further exemplified by the comparable 3D and 96-well ELISA results obtained in this study. The simplicity offered by 3D printing applications in the fields of microfluidics, LOC technologies and clinical laboratory medicine diagnostics [20,21,22], and the improved antibody detection sensitivity even using very diluted samples as noted in our study provides future opportunities for readjustments to be made, including potentially miniaturization to reduce time and amount of reagents needed, reducing cost including increasing portability of such ELISA-based diagnostics. The impact of these technologies in the use of ELISA systems for the rapid and accurate pathogen identification and serological diagnosis of infectious diseases such as in monitoring of infectious diseases during disease outbreaks or in the care of individual patients and its potential to reduce cost and time are crucial factors to consider especially in resource-constraint settings [10,27,28]. Additionally, the commonalities of principles and applications shared by the different disciplines such as health, food industry, environmental, chemistry, biomedical and engineering and the flexibility in designing-product development-feedback cycle offered through 3D printing platforms provide future opportunities to be explored [20].

## 5. Conclusions

The ease and rapidity of design development and the high achievable accuracy of one-step fabrication technologies such as 3D printing in the rapid prototyping of 3D wells using readily available 3D printing platforms and polymer materials provided an opportunity for the rapid diagnostic performance of a 3D well ELISA in infectious diseases to be performed in this study. The 3D wells developed in this study showed an improved detection sensitivity of up to 2.25 folds higher compared to 96-well ELISA. This study provides room for expansion of this technology towards miniaturization to reduce the time and volume of reagents required in immunological diagnosis for infectious diseases and its application in other disciplines.
